# The Injection of miR-1/133 Antagonist Notably Accelerated the Healing Process of Exercise-Induced Skeletal Muscle Injury

**DOI:** 10.33549/physiolres.935529

**Published:** 2026-06-01

**Authors:** Xiujuan LIU, Meng YUAN, Nianyun ZHANG, Jing XING

**Affiliations:** 1 Department of Sports and Health, Nanjing Sports Institute, Nanjing, China; 2 Jiangsu Collaborative Innovation Center for Sport and Health Project, Nanjing, China; 3 Department Science Experiment Center, Nanjing Sports Institute, Nanjing, China

**Keywords:** Exercise, Muscle damage, Skeletal muscle regeneration, MRTF-A, Pax7, SRF

## Abstract

Exercise-induced muscle damage (EIMD) significantly impacts daily work and life. The rapid promotion of repair for EIMD is worthy of attention. This study aimed to investigate the effect and mechanism of microRNAs in treating EIMD. By establishing an acute skeletal muscle injury model, we determined the key time point for skeletal muscle injury repair and the time-specific changes in MRTF-A/Pax7/SRF and muscle regeneration factors during the repair process. MicroRNAs antagonists were injected to verify the targeting relationship between miR-1/133a and MRTF-A/Pax7/SRF. A single bout of acute eccentric exercise caused significant damage to the morphological ultrastructure of rat gastrocnemius muscles, with the most severe injuries occurring 72 h after exercise. At this particular time point, it was identified as crucial for damage repair. Both miR-1-3p and miR-133a-3p collectively targeted and suppressed the protein translation of MRTF-A, Pax7, and SRF. Furthermore, both miR-1-3p and miR-133a-3p antagonists targeted the MRTF-A/Pax7 axis as well as the MRTF-A/SRF axis. MiR-1-3p antagonists primarily promote muscle proliferation and differentiation, while miR-133a-3p antagonists mainly promote differentiation while inhibiting atrophy. Combined injection effectively promote both muscle proliferation and differentiation while inhibiting atrophy, thereby facilitating damage repair in skeletal muscle fiber structure.

## Introduction

Exercise-induced muscle damage (EIMD) is more likely to occur in people who do not regularly exercise after high-intensity or one-time high-intensity exercise, most of which involve a large number of muscle eccentric contraction processes [1]. A series of muscle fiber damage and inflammation induced by EIMD [2] and delayed onset muscle soreness can significantly disrupt daily life, subsequently dampening people’s motivation for exercise. Therefore, how to rapidly promote the repair of EIMD has become the focus of sports science research.

Skeletal muscle satellite cells (SCs) are the core cells responsible for the growth and regeneration of skeletal muscle. Paired box 7 (Pax7) is the key regulatory factor for the activation and proliferation of muscle satellite cells. Pax7 plays a key regulatory role in the development, growth, and regeneration of skeletal muscle. As the upstream regulator of myogenic regulatory factors (MRFs) [3], Pax7 promotes the activation and proliferation of muscle satellite cells while inhibiting their differentiation. And maintain the role of satellite cell self-renewal [4] to meet the functional requirements of muscle tissue growth and regeneration. When skeletal muscle is damaged, the expression of Pax7 significantly increases to promote the activation of muscle satellite cells and rapidly up-regulate the expression levels of downstream Myod and Myf5, further inducing skeletal muscle proliferation [5].

Serum response factor (SRF) is a member of the transcriptional activator MADS (MCM1, agamous, SRF) family. Its significance in skeletal muscle growth, development, injury, and regeneration should not be overlooked. SRF mainly promotes the expression of downstream differentiation factors in skeletal muscle and negatively regulates the proliferation of muscle cells to induce the process of muscle regeneration [6]. Recent studies have found that Myocardin-Related Transcription Factor A (MRTF-A), a synergistic factor of SRF, is essential for the development and maintenance of skeletal muscle [7] and is widely expressed in muscle satellite cells. MRTF-A is a newly discovered SRF synergistic factor found in skeletal muscle satellite cells, which are crucial for muscle regeneration [8]. As the upstream regulator of SRF, MRTF plays a core role in the regulation of actin expression and cytoskeletal dynamics [7]. Its activity can be effectively enhanced by mediating SRF to regulate the myogenic differentiation of skeletal muscle [9]. The MRTF-A/SRF axis can not only activate differentiation by promoting MyoD expression but also stimulate the differentiation of skeletal muscle. MyoD can also enhance the expression of downstream cell cycle arrest genes, thereby inhibiting cell proliferation [10]. Studies have found that MRTF-A plays a crucial role in the process of skeletal muscle injury and atrophy [7,10]. The loss of MRTF-A can lead to excessive proliferation and abnormal differentiation of satellite cells, hindering the regeneration of skeletal muscle after injury. However, there are few reports on the correlation between MRTF-A mediating SRF and Pax7 in the regeneration process of exercise-induced skeletal muscle injury.

MicroRNAs (miRNAs) are short, non-coding small RNA molecules that regulate gene expression by binding to the 3′-UTR of target messenger RNA (mRNA) for translational inhibition or degradation of mRNA [11]. miRNAs are involved in many regulatory processes of skeletal muscle, including myogenesis, myoblast proliferation and differentiation, regeneration. Pax7 is a key regulator of muscle regeneration and an important target of miRNA regulation [12]. miR-1, miR-133a/b, and miR-206 are common microRNAs studied for their role in regulating skeletal muscle injury and regeneration by targeting Pax7 [13]. Their regulatory effects on Pax7 have a significant impact on the repair of skeletal muscle injuries [14–16]. SRF is also targeted by miRNA and collaborates with other factors to regulate the expression of downstream myogenic factors [17]. At present, it has been verified that miR-133a and miR-133b can target and regulate SRF, which plays a role in the proliferation and differentiation of muscle cells mainly by silencing the expression of the SRF gene. They promote the proliferation of myoblasts [18] and inhibit the expression of muscle differentiation factors [13]. Current studies on the targeted regulation of MRTF-A by miRNA mainly focus on cancer and fibrosis [19]. While limited research has been conducted on its regulation in skeletal muscle.

Therefore, this research established a skeletal muscle injury model through one-time eccentric exercise, observed the changes of MRTF-A, Pax7, SRF, miR-1 and miR-133 at various time points during skeletal muscle regeneration and repair after eccentric exercise, and verified whether miR-1 and miR-133 targeted the regulation of MRTF-A/Pax7/SRF in order to identify potential interventions for promoting rapid repair of exercise-induced skeletal muscle injury.

## Materials and Methods

### Experimental animals

A total of sixty-six 8-week-old male SD rats were selected from Shanghai Slack Laboratory Animal Co., LTD. After a week of adaptation, the rats were kept in a controlled environment with a temperature of 23±2 °C, humidity at 50±10 %, free access to water, and a light-dark cycle of 12 h:12 h. Following the adaptation period, forty-two rats were randomly assigned to either the sedentary control group (group C, n=6) or the exercise group (group E, n=36). The sedentary control group was allowed free movement while the exercise group underwent acute eccentric exercise. The exercise group was further divided into six subgroups with six rats in each subgroup: immediately after exercise (E0), 12 h after exercise (E12), 24 h after exercise (E24), 48 h after exercise (E48), 72 h after exercise (E72), and one week after exercise (E1w). Twenty-four rats underwent acute eccentricity exercises and were randomly allocated to NC group, miR-1 antagomir group, miR-133 antagomir group and miR-1+133 antagomir group. This study was approved by the Experimental Animal Ethics Committee of Nanjing Sport Institute (DW-2023003) and all procedures complied with China’s animal management regulations as well as international ethical standards [20].

### Exercise program

**Fig. 1:**
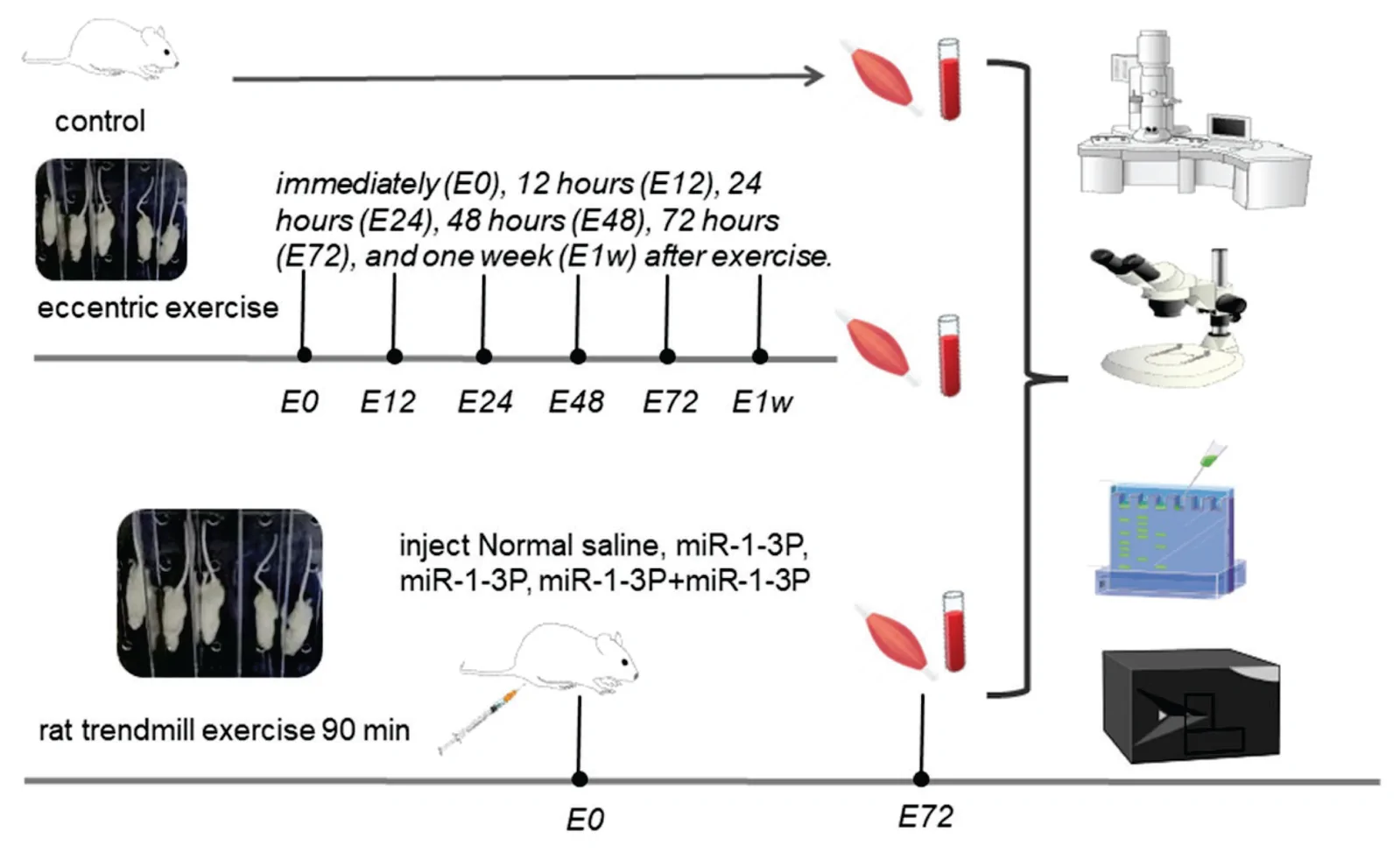
Schematic diagram of experimental design.

The rats were subjected to a one-time eccentric according to the previous scheme [21]. Subsequently, they were intraperitoneally injected with 1 ml/100 g Ethyl carbamate immediately after exercise, as well as at 12 h, 24 h, 48 h, 72 h, and one week post-exercise. Blood and gastrocnemius tissue samples were then collected for subsequent analysis. The miRNA antagomirs (NC group, miR-1 Antagomir group, miR-133 Antagomir group, and miR-1+133 Antagomir group) were injected into the right gastrocnemius muscle immediately after exercise with a concentration of 20 μM and a volume of 40 μl. The miRNA antagomirs were purchased from Guangzhou Ruibo Biotechnology Co., Ltd. The NC group received an injection of an equivalent volume of normal saline. After injection completion, the rats were housed individually until three days post-injection when blood and gastrocnemius tissue samples were collected under anesthesia for follow-up testing. The experimental design is shown in Figure 1.

### Serum creatine kinase (CK) detection

The blood samples were obtained from the abdominal aorta of the rats. After a 20-minute rest, the samples were centrifuged at 3000 rpm at 4 °C for 15 min to separate serum. The levels of serum CK were measured using an automatic biochemical analyzer (Cat. No. 7100, HITACHI, Japan).

### Ultrastructure observation of gastrocnemius muscle

The gastrocnemius muscle tissue was dissected into slender muscle bundles (approximately 1 mm × 1 mm × 5 mm) along the longitudinal axis of the muscle fibers using a sharp, sterile double-edged blade. Care was taken to avoid mechanical compression or traction during handling. The excised muscle bundles were immediately immersed in a pre-cooled (4 °C) fixative solution containing 2.5–4 % glutaraldehyde. While submerged in the fixative, both ends of the tissue blocks were further trimmed with a sharp blade to ensure uniformity and then sectioned into smaller fragments of approximately 1 mm^3^. These tissue samples were transferred into centrifuge tubes containing fresh glutaraldehyde fixative and fixed overnight at 4 °C. Subsequently, the tissue blocks were rinsed three times with 0.1 M phosphate buffer (pH 7.4), 15 min per rinse, to thoroughly remove residual glutaraldehyde and prevent potential precipitation during subsequent osmium tetroxide treatment. Next, the tissue blocks were post-fixed in 1 % osmium tetroxide (OsO_4_) aqueous solution for 1–2 h at 4 °C under dark conditions. Following fixation, the samples were rinsed three times with double-distilled water (15 minutes each) to eliminate excess osmium tetroxide. Dehydration was carried out through an ascending ethanol series: 30 %, 50 %, 70 %, 80 %, 90 %, and 100 %, with each step lasting 15–20 min. The initial stages up to 70 % ethanol were performed at 4 °C, after which the samples were equilibrated to room temperature. Thereafter, the tissue blocks were transitioned gradually into epoxy resin (Epon 812) *via* a series of mixtures of dehydrating agent and embedding resin: ratio 3:1 (v/v, 1–2 h, room temperature), 1:1 (2–4 h or overnight), 1:3 (2–4 h), and finally pure resin (2–4 h or overnight). Once fully infiltrated, the tissue blocks were placed into embedding molds filled with freshly prepared resin. Using fine forceps, each block was carefully positioned at the center of the mold base and oriented appropriately. The molds were then placed in an oven and polymerized under controlled temperature conditions: 37 °C for 12 h, followed by 45 °C for 12 h, and finally 60 °C for 24–48 h. After complete polymerization, the hardened resin blocks were removed from the molds. The top surface of each block was trimmed with a single-edged blade to form a small, smooth trapezoidal face, exposing the central region of the embedded tissue. Semi-thin sections (0.5–1 μm thick) were cut using a glass knife, stained with toluidine blue, and examined under a light microscope to confirm the orientation of muscle fibers and identify the target area for ultrastructural analysis. The block face was further refined using a trimming knife to achieve a final surface area of less than 0.5 mm × 0.5 mm, ensuring that the opposing sides were parallel and smoothly finished. The trimmed block was mounted on an ultramicrotome, and ultrathin sections (50–90 nm thick) were collected onto a water-filled trough. A fine brush was used to flatten the ribbon of sections on the water surface. Copper grids pre-coated with support film were carefully inserted beneath the floating ribbon from below the water surface, gently lifted, and excess moisture was absorbed using filter paper. The grids carrying the sections were then floated on a saturated aqueous solution of uranyl acetate and stained in the dark for 15–30 min. After thorough rinsing with double-distilled water, the grids were transferred to freshly prepared lead citrate staining solution and incubated in a desiccator containing NaOH pellets for 5–10 min to prevent carbon dioxide interference. The filter paper was thoroughly rinsed with double-distilled water and subsequently dried. Following staining, the copper grid was placed in a designated storage container and kept in a desiccator. Imaging was performed using a dedicated imaging system for transmission electron microscopy (830.10U3, Gatan, USA), and morphological observation was conducted using a transmission electron microscope (JEM-1400, JEOL, Japan).

### Immunofluorescence staining

The gastrocnemius muscle samples were embedded in OCT and placed in a frozen microtome (Cat. No.CM1950, LEICA, Germany) for sectioning at a thickness of 10 μm. The sections were then fixed with 4 % paraformaldehyde, rinsed with distilled water, blocked with goat serum, and incubated in a humidified chamber for 1 h. After removing the blocking solution, primary antibodies Pax7 and Laminin were applied and the sections were incubated overnight at 4 °C in a refrigerator (see Table 1 for antibody details). The next day, the sections were washed three times with PBS after removal of the primary antibodies. Subsequently, secondary Anti-mouse IgG (H+L) Alexa Fluor®555 Conjugate and Anti-rabbit IgG (H+L) Alexa Fluor®488 Conjugate were applied before incubating the sections in a humidified chamber at a constant temperature of 37 °C for one hour (refer to Table 1 for detailed antibody information). After removing the secondary antibody solution, the sections were washed three times with PBS before being mounted with an anti-fade mounting medium containing DAPI. Finally, images were captured under a fluorescence microscope (Zeiss, Imager.A2, Germany) at a magnification of 200×. The number of satellite cells in each image was quantified using image analysis software (ImageJ).

### Muscle protein extraction and Western blot detection

Protein extraction was performed from tissue samples. Briefly, 50 mg of tissue was homogenized on ice in 500 μl of ice-cold RIPA lysis buffer supplemented with protease inhibitors, phosphatase inhibitors, and EDTA using a grinding tube containing ceramic beads. The homogenate was incubated on ice for 10 min and then centrifuged at 12,000 rpm for 10 min at 4 °C. The resulting supernatant was transferred to a new 1.5 ml microcentrifuge tube and maintained on ice. Protein concentration was determined using the bicinchoninic acid (BCA) assay. For immunoblotting, proteins were separated by SDS-PAGE on 7.5 % or 10 % polyacrylamide gels prepared with a rapid gel kit. Equal amounts of protein from each sample, along with 5 μl of a protein molecular weight marker, were loaded into the wells. Following electrophoresis, proteins were transferred onto a PVDF membrane. The membrane was blocked with blocking buffer and subsequently washed three times with TBST. The membrane was then cut according to the molecular weights of the target and internal reference proteins. Membrane strips were incubated overnight at 4 °C with specific primary antibodies diluted as specified in Table 1. After three washes with TBST, the membranes were incubated with appropriate horseradish peroxidase (HRP)-conjugated secondary antibodies (as indicated in Table 1) for 1 h and 30 min at room temperature. Following an additional series of TBST washes, protein bands were visualized using a chemiluminescent substrate and imaged using a chemiluminescence imaging system. Finally, band intensities were quantified by analyzing gray values using ImageJ software for statistical evaluation.

### Bioinformatics prediction

**Fig. 2:**
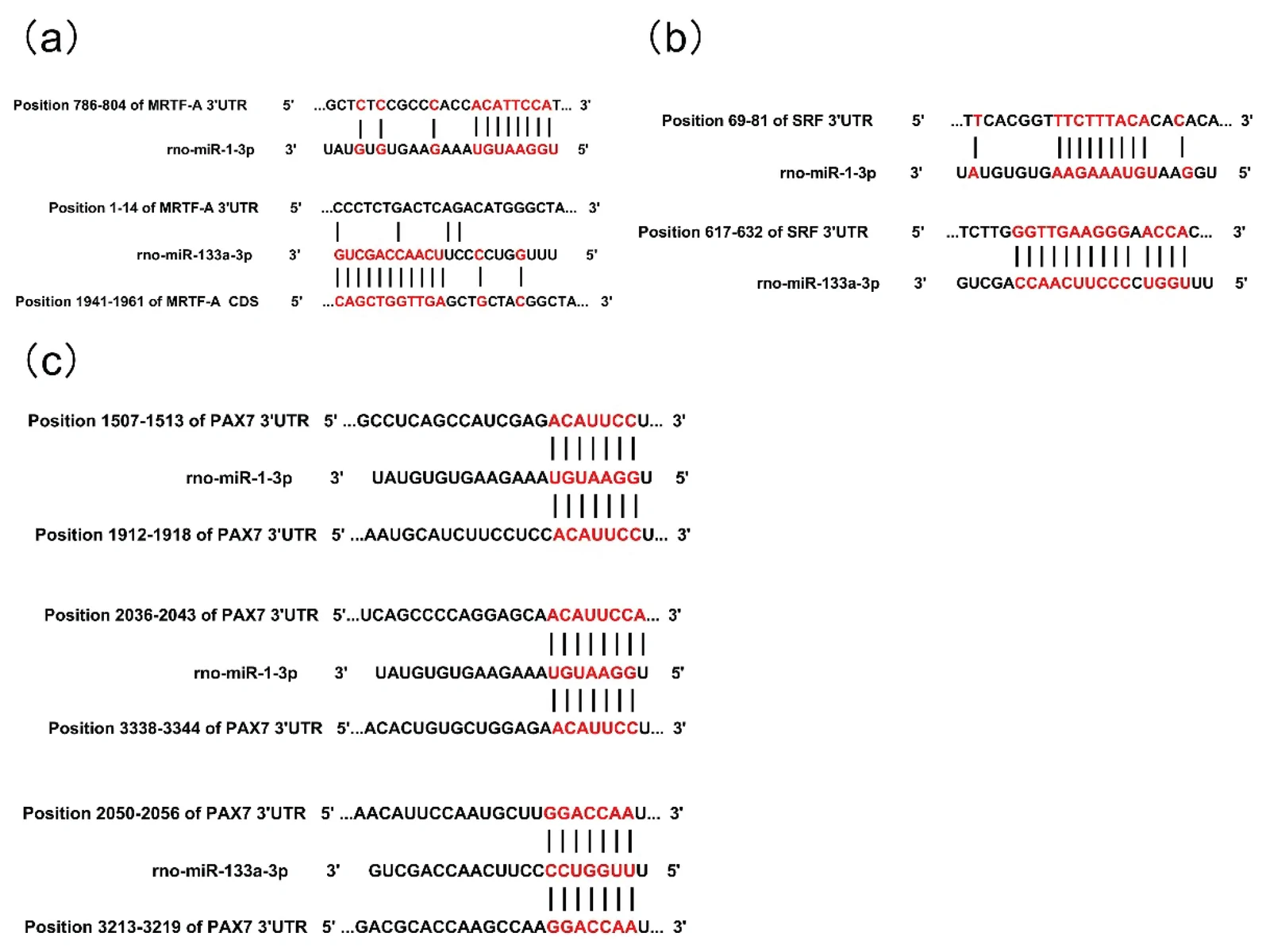
Predicted binding sites of miR-1-3p/miR-133a-3p to target genes were identified. (**a**) Primer Premier 5 software detected the gene complementary pairing binding sites of miR-1-3p/miR-133a-3p and MRTF-A; (**b**) Primer Premier 5 software detected the gene complementary pairing binding sites of miR-1-3p/miR-133a-3p and SRF; (**c**) TargetScan database predicted the binding site of miR-1-3p/miR-133a-3p to the target gene Pax7.

Utilize the Targetscan database (https://www.targetscan.org/vert_72/) and Primer Premier 5 software to predict the potential binding sites of miR-1–3p and miR-133-A-3p on the target genes Pax7, SRF, and MRTF-A in rats (Fig. 2).

### mRNA extraction and real-time fluorescence quantitative PCR detection

Total RNA was extracted from gastrocnemius tissue utilizing TRIzol RNA extraction TaKaRa RNA Extraction Kit (Takara Biomedical Technology Co., Ltd.) in accordance with the manufacturer’s protocol. The purity and concentration of the total RNA from each sample were assessed using a Nanodrop spectrophoto-meter, while RNA quality was evaluated through agarose gel electrophoresis. mRNA detection using PrimeScript™ II High Fidelity RT-PCR Kit (Takara Biomedical Technology Co., Ltd.) in accordance with the manufacturer’s protocol. miRNA detection using and Mir-X miRNA qRT-PCR TB Green® Kit (Takara Biomedical Technology Co., Ltd.) in accordance with the manufacturer’s protocol. Primers for target genes and beta-actin were designed using Primer Premier 5 software and synthesized by Shanghai Jierui Biotechnology Co., LTD (Table 2) for real-time fluorescence quantitative PCR analysis.

### Data statistics

Statistical analysis was performed using GraphPad Prism 7 software to conduct one-way ANOVA, and the experimental data were presented as mean ± standard error (SEM). A difference was considered significant at P<0.05, and highly significant at P<0.01.

## Results

### The ultrastructure of the gastrocnemius muscle and the population of satellite cells analysis treated with microRNA antagomir

**Fig. 3:**
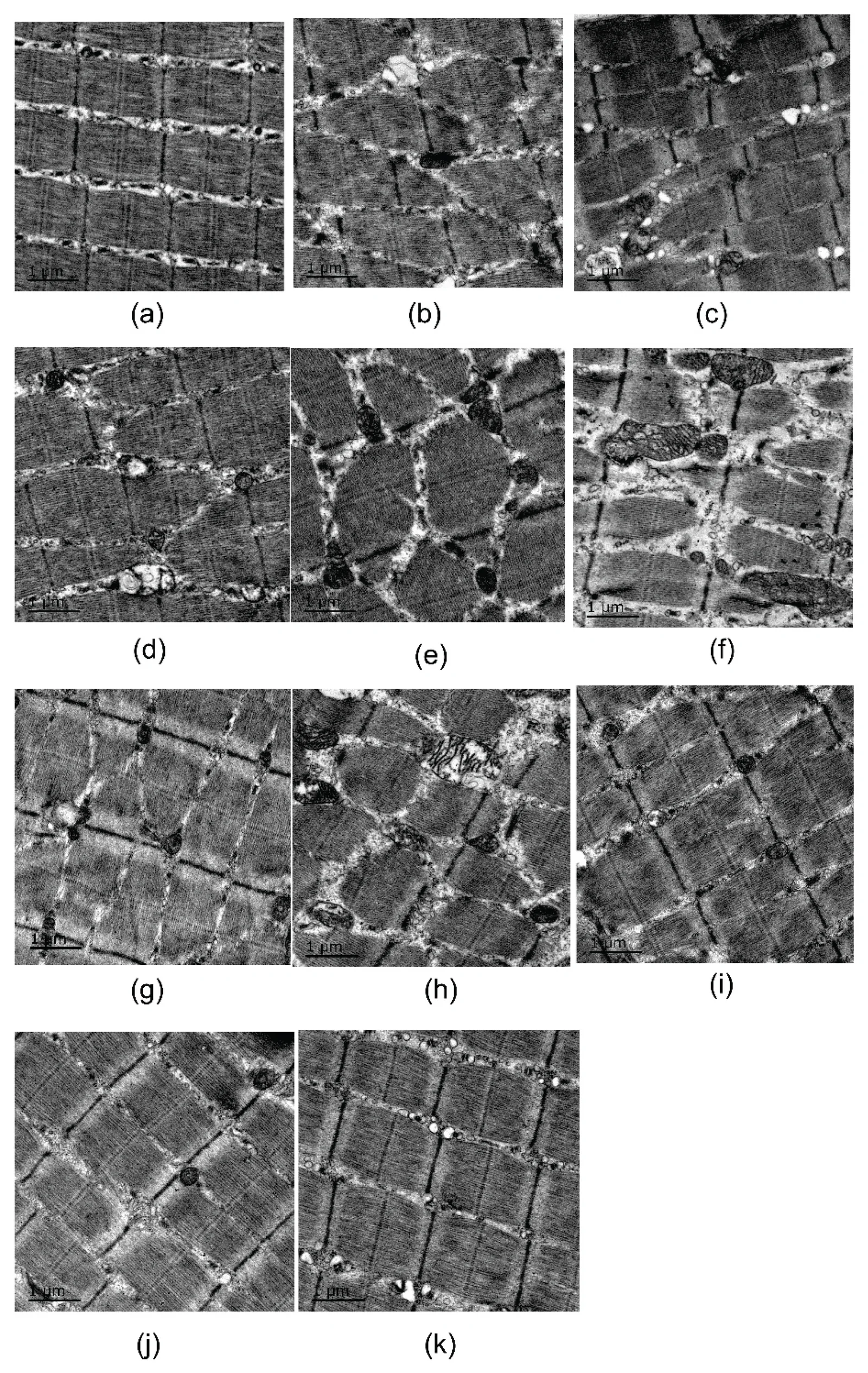
Ultrastructure of gastro-cnemius muscle of rats in each group. Magnification, 2000 times, scale, 1 μm. (**a**) sedentary control group; (**b**) E0, immediately after exercise group; (**c**) E12, 12 h after exercise group; (**d**) E24, 24 h after exercise group; (**e**) E48, 48 h after exercise group; (**f**) E72, 72 h after exercise group; (**g**) E1w, one week after exercise group; (**h**) NC group; (**i**) miR-1 Antagomir group; (**j**) miR-133 Antagomir group; (**k**) miR-1+133 Antagomir group.

Myofibrillary rupture was observed immediately following exercise, characterized by irregular Z-line alignment and mitochondrial swelling (Fig. 3b). At 12 h post-exercise, the Z-line arrangement remained disorganized, accompanied by abnormal mitochondrial morphology (Fig. 3c). By 24 h post-exercise, individual Z-lines exhibited marked disarray, while mitochondria displayed pronounced swelling and vacuolation (Fig. 3d). At 48 h post-exercise, Z-lines appeared blurred, and mitochondrial abnormalities persisted (Fig. 3e). At 72 h post-exercise, Z-line disruption was most evident, with mitochondrial swelling reaching its maximum extent (Fig. 3f). One week after exercise, Z-lines gradually regained structural clarity, and no significant mitochondrial abnormalities were detected (Fig. 3g). In contrast, in the miR-1 antagomir and miR-133 antagomir groups, myofibrils were well-organized, with orderly Z-line arrangements and largely preserved mitochondrial morphology, exhibiting only mild oval-shaped swelling (Fig. 3i, j). Notably, in the miR-1+133 antagomir group, Z-lines were regularly aligned with clearly discernible light and dark bands, the overall sarcomeric structure remained intact, sarcoplasmic reticulum swelling was minimal, and no apparent mitochondrial alterations were observed (Fig. 3k). These findings suggest that administration of miR-1-3p and miR-133a-3p antagonists effectively mitigates skeletal muscle fiber damage induced by acute eccentric exercise.

**Fig. 4:**
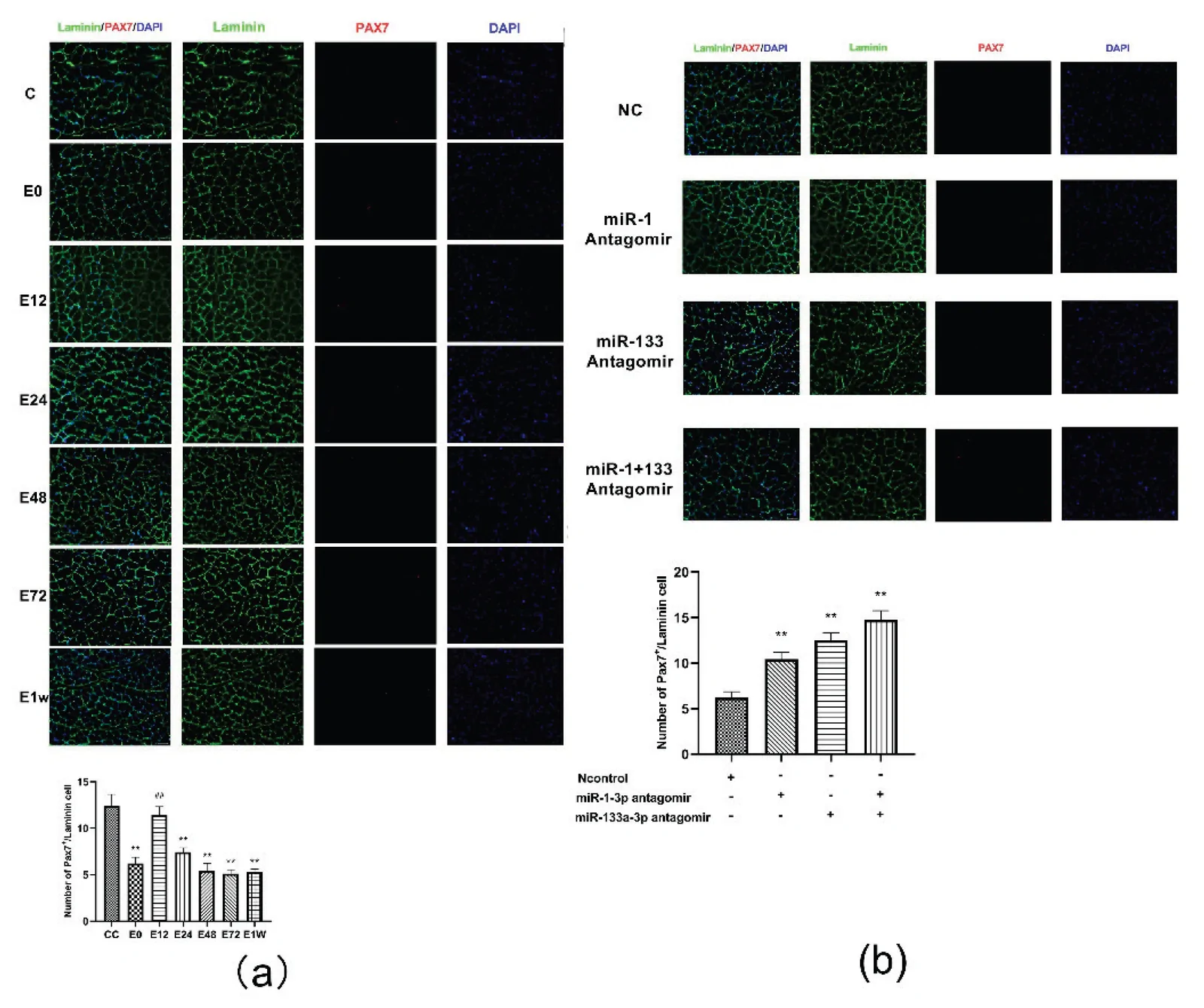
Changes in the number of satellite cells in gastrocnemius muscle of rats in each group. Image magnification: 200 times; Scale: 50 μm. immunofluorescence staining of gastrocnemius muscle satellite cells in each group. Pax7 (red) indicates Pax7-positive cells; Laminin (green) indicates laminin, which is the boundary of the fiber; DAPI (blue) represents the nucleus. (**a**), the number of Pax7/Laminin co-staining positive cells in each group at different time points after eccentric exercise; (**b**) the number of Pax7/Laminin co-staining positive cells in each group were injected with miRNA antagonists; compared to group C/NC, * P<0.05, ** P<0.01; Compared to E0 group, ^#^ P<0.05, ^##^ P<0.01.

We observed the number of muscle satellite cells in the gastrocnemius muscle was significantly decreased in the E0, E24, E48, E72 and E1w groups (P<0.01) (Fig. 4a). Additionally, there was a significant increase in the number of muscle satellite cells in the E12 group compared to the E0 group (P<0.01) (Fig. 4a). The satellite cell count showed a significant increase following the injection of significantly increased after miR-1 antagomir, miR-133 antagomir, and miR-1+133 antagomir administration (P<0.01) (Fig. 4b).

### Creatine kinase (CK) levels analysis in serum treated with microRNA antagomir

**Fig. 5:**
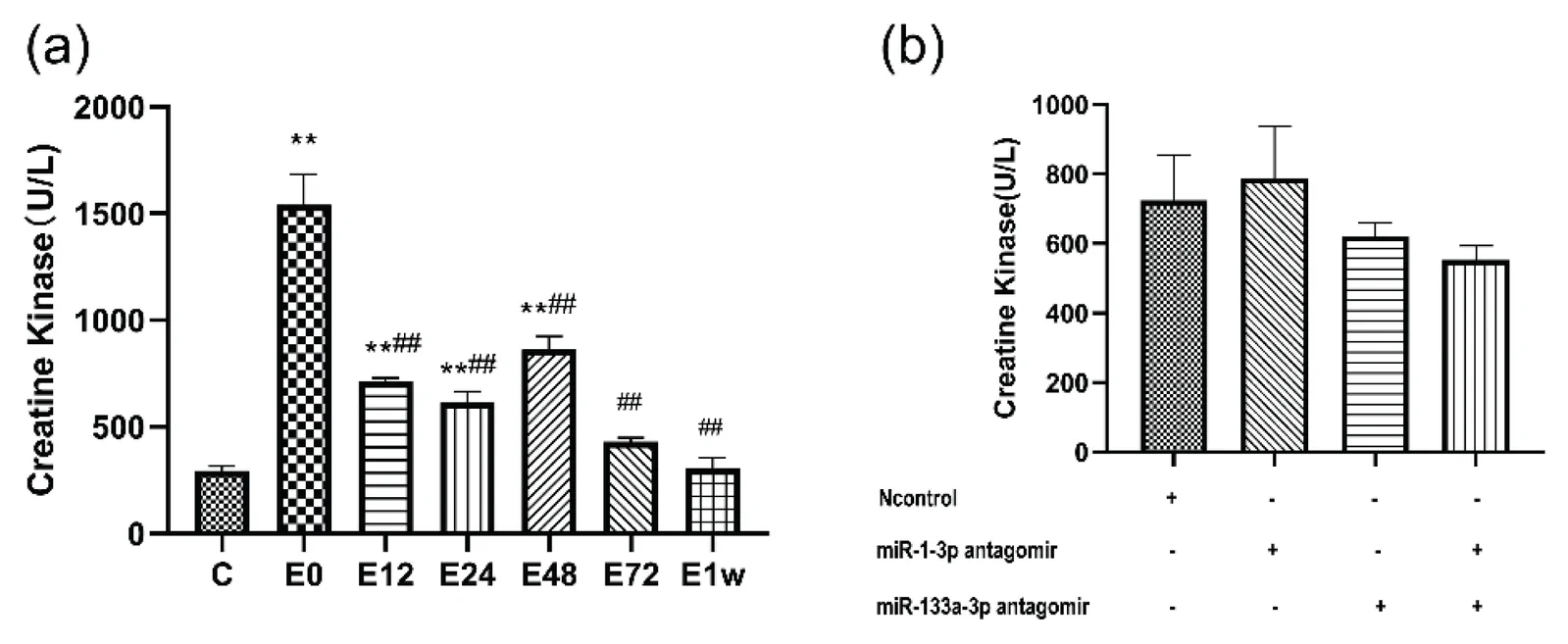
Serum creatine kinase (CK) content of rats in each group (**a**) Serum CK content in each group at different time points after eccentric exercise; (**b**) Serum CK content in each group were injected with miRNA antagonists; compared to group C, * P<0.05, ** P<0.01; Compared to E0 group, ^#^ P<0.05, ^##^ P<0.01.

Compared to group C, the serum CK levels of rats in the E0, E12, E24, and E48 groups were significantly increased. However, there was no significant change in the serum CK levels of rats in the E72 and E1w groups (Fig. 5a). Additionally, there were no signific ant differences in serum CK levels between the miR-1 antagomir group, miR-133 antagomir group, and miR-1+133 antagomir group compared to the NC group (Fig. 5b).

### mRNA expression analysis of miR-1-3p/miR-133a-3p in the gastrocnemius muscle of rats treated with microRNA antagomir

**Fig. 6:**
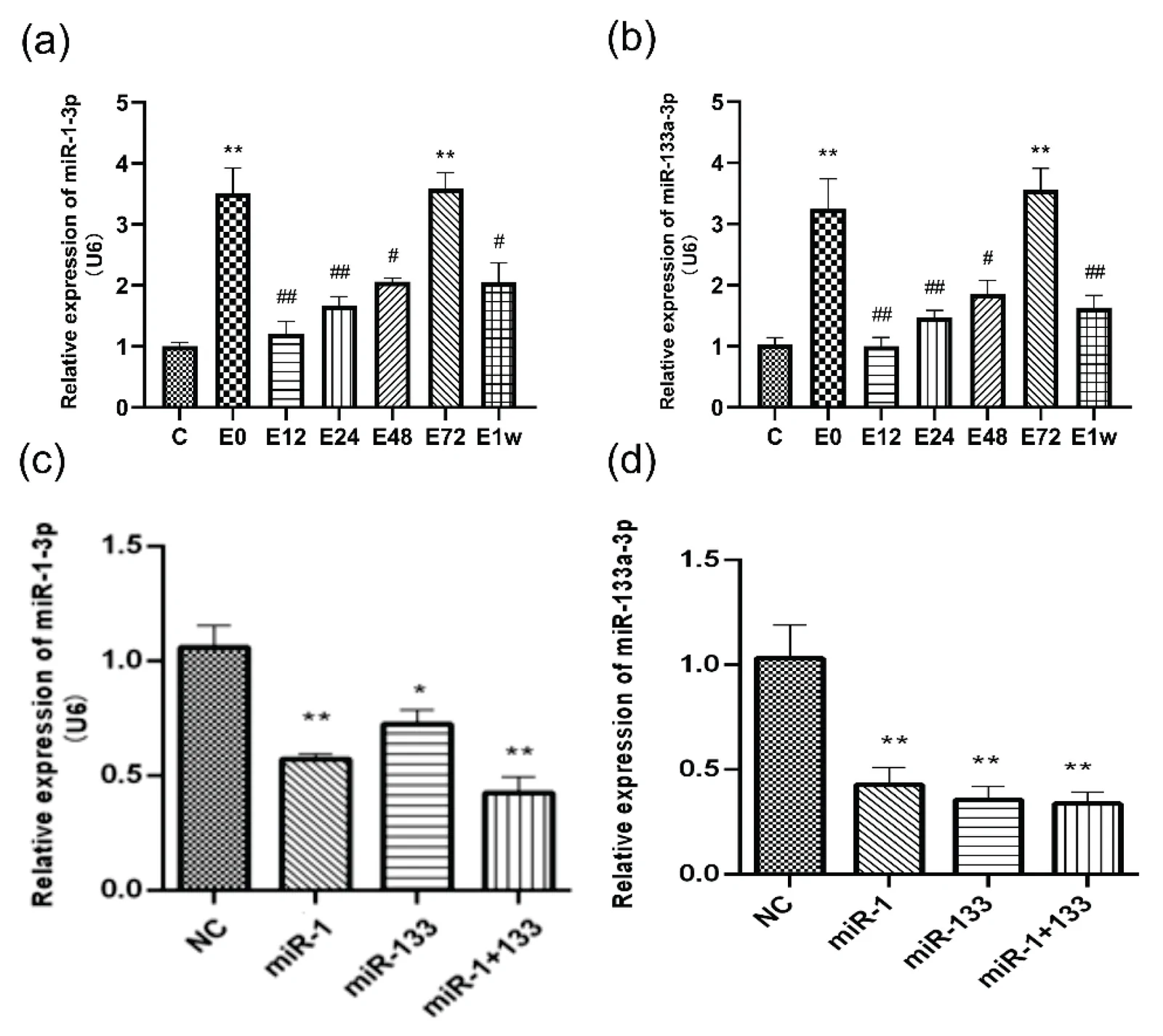
Changes in the expression of miR-1-3p/miR-133a-3p in the gastrocnemius muscle of rats in each experimental group. (**a**) and (**b**) the expression of miR-1-3p/miR-133a-3p in each group at different time points after eccentric exercise; (**c**) and (**d**) the expression of miR-1-3p/miR-133a-3p in each group were injected with miRNA antagonists; compared to group C/NC, * P<0.05, ** P<0.01; Compared to E0 group, ^#^ P<0.05, ^##^ P<0.01.

The expressions of miR-1-3p and miR-133a-3p in groups E0 and E72 were significantly upregulated compared to group C (P<0.05) (Fig. 6a, b). Following injection of miR-1 antagomir, miR-133 antagomir, and miR-1+133 antagomir, the expressions of miR-1-3p/miR-133a-3p were significantly downregulated (P<0.05) (Fig. 6c, d).

### mRNA and protein expression analysis of MARTF-A, Pax7, and SRF treated with microRNA antagomir

**Fig. 7:**
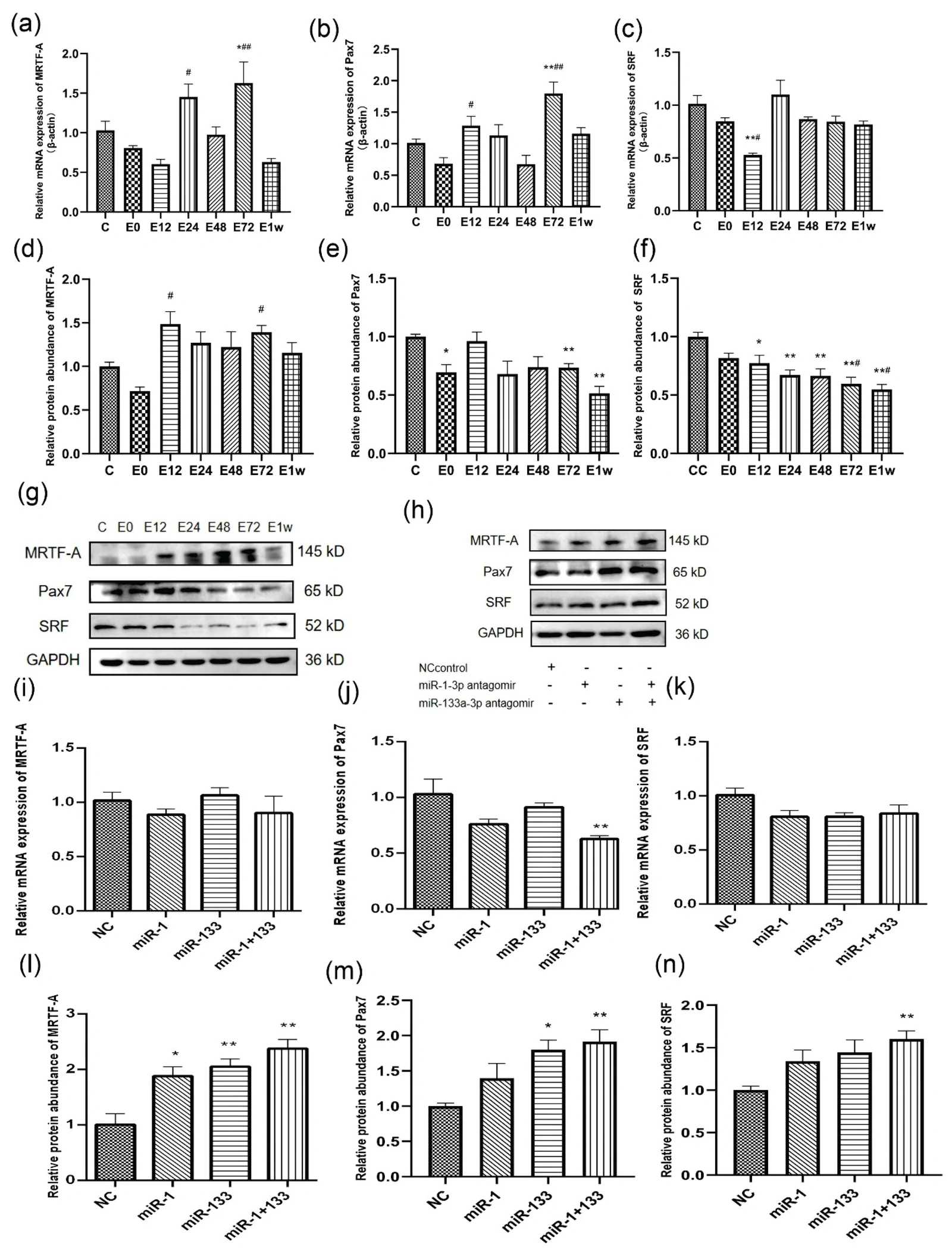
mRNA and protein expression of MRTF-A/Pax7/SRF in gastrocnemius muscle of rats in each group. (**a–f**) mRNA and protein expression of MRTF-A/Pax7/SRF in gastrocnemius muscle of rats in each group at different time points after eccentric exercise; (**i–n**) mRNA and protein expression of MRTF-A/Pax7/SRF in gastrocnemius muscle of rats in each group were injected with miRNA antagonists; (**g–h**) MRTF-A/Pax7/SRF protein western blot bands; compared to group C/NC, * P<0.05, ** P<0.01; Compared to E0 group, ^#^ P<0.05, ^##^ P<0.01.

Compared to group C, the mRNA expressions of MARTF-A and Pax7 in group E72 showed a significant increase (P<0.05), while the mRNA expressions of SRF in group E12 exhibited a significant decrease (P<0.05). In comparison to the E0 group, the mRNA expressions of MARTF-A in the E24 and E72 groups were significantly elevated (P<0.05), with Pax7 mRNA expression also showing a significant increase in the E12 and E72 groups (P<0.05). The SRF mRNA level in the E12 group experienced a significant decrease (P<0.01) (Fig. 7a, c, e). Compared to group C, both Pax7 and SRF protein expressions were significantly reduced in groups E72 and E1w (P<0.05), with SRF protein expressions also experiencing a significant decrease in groups E12, E24 and E48 (P<0.05). However, MRTF-A protein expression did not show any significant change (P>0.05). In comparison to the E0 group, the MRTF-A protein expressions in the E12 and E72 groups showed a significant increase (P<0.05), while the SRF protein expression in the E72 and E1w groups experienced a significant decrease (P<0.05) (Fig. 7b, d, f). There were no statistically significant differences in the expression of MRTF-A mRNA and SRF mRNA among all groups following injection of miR-1 antagomir, miR-133 antagomir, and miR-1+133 antagomir (P>0.05). The expression of MRTF-A protein was significantly increased in the miR-1 antagomir, miR-133 antagomir group and miR-1+133 antagomir group (P<0.05) (Fig. 7j). The expression of SRF protein was significantly increased in the miR-1+133 antagomir group (P<0.01) (Fig. 7n). Additionally, Pax7 mRNA expression was significantly decreased in the miR-1+133 antagomir group (P<0.01) (Fig. 7k), while the expression of Pax7 protein was significantly increased in the miR-133 antagomir group and miR-1+133 antagomir group (P<0.01) (Fig. 7l). This suggests that injection of miR-133 antagonists can selectively promote Pax7 protein upregulation and increase muscle satellite cell numbers after acute eccentric exercise, thereby initiating a repair response and accelerating myofiber regeneration.

### Protein expression analysis of cell proliferation, differentiation, and atrophy in gastrocnemius muscle treated with microRNA antagomir

**Fig. 8:**
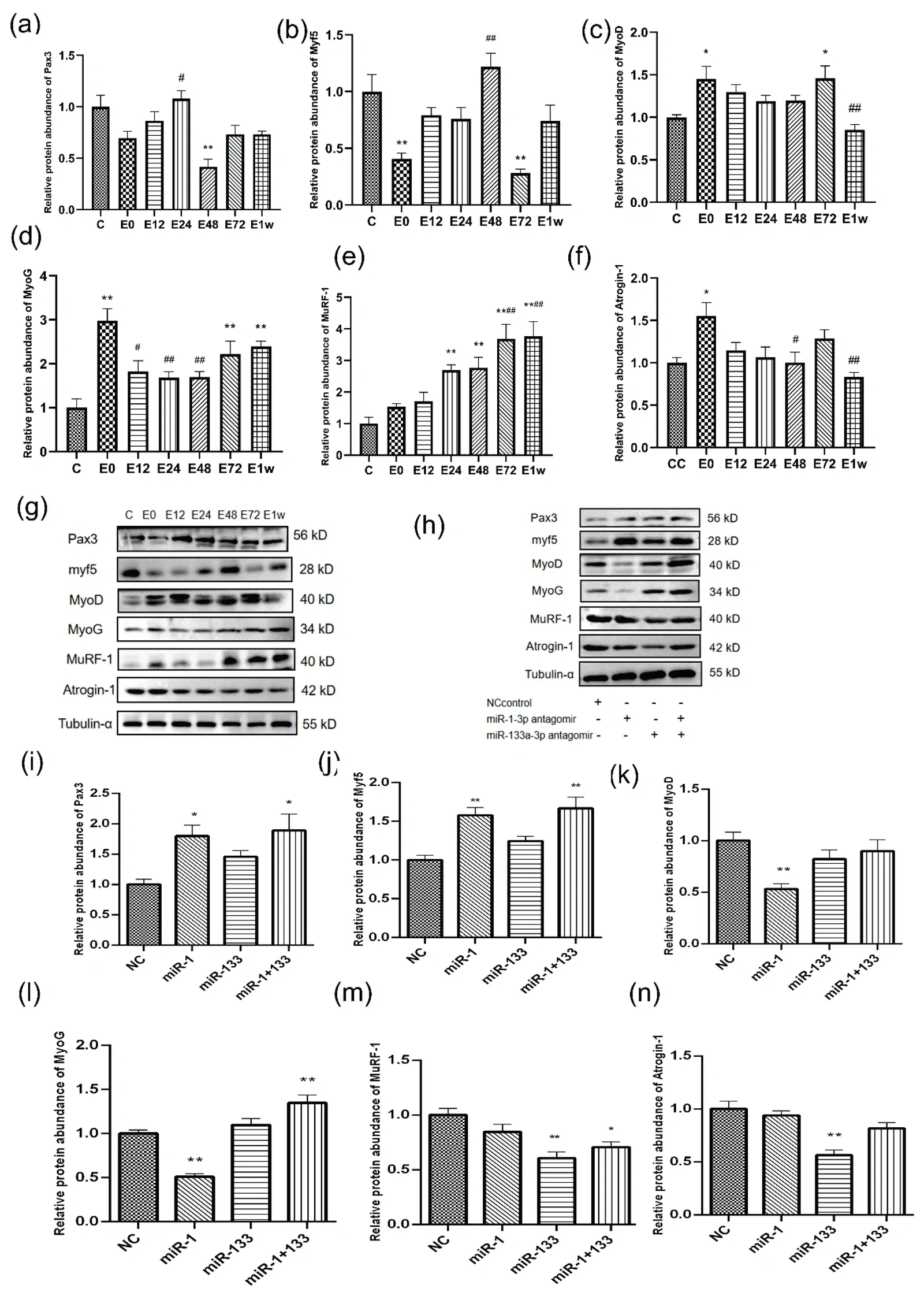
The expression of protein in gastrocnemius muscle of rats in each group. (**a–f**) protein expression of Pax3, myf5, MyoD, MyoG, MuRF-1, Atrogin-1 in gastrocnemius muscle of rats in each group at different time points after eccentric exercise; (**i–n**) protein expression of Pax3, myf5, MyoD, MyoG, MuRF-1, Atrogin-1 in gastrocnemius muscle of rats in each group were injected with miRNA antagonists; (**g–h**) protein western blot bands; compared to group C/NC, * P<0.05, ** P<0.01; Compared to E0 group, ^#^ P<0.05, ^##^ P<0.01.

Compared to group C, the expression of Pax3 protein was significantly reduced in the E48 group (P<0.05), while Myf5 protein expression was significantly decreased in the E0 and E72 groups (P<0.01). In comparison to the E0 group, Pax3 protein expression was significantly increased in the E24 group (P<0.05), and Myf5 protein expression was extremely significantly increased in the E48 group (P<0.01) (Fig. 8a, b). The expression of MyoD protein was significantly increased in groups E0 and E72 compared to group C (P<0.05), while the expression of MyoG protein exhibited a significant increase beginning at E0 (P<0.05). The expression of MyoD protein was significantly decreased in the E1w group compared to the E0 group (P<0.05) (Fig. 8c, d). The expression of MuRF-1 protein exhibited a significant increase beginning at E24 compared to group C (P<0.05). In comparison to the E0 group, MuRF-1 protein expression was significantly increased in the E72 group and E1w (P<0.01), while atrogin-1 protein in groups E48 and E1w was significantly decreased (P<0.05) (Fig. 8e, f). After the administration of miR-1 antagomir, there was a significant increase in Pax3 and myf5 protein expressions (P<0.05) (Fig. 8i, j), while MyoD protein levels showed a significant decrease (P<0.05) (Fig. 8k). Following the injection of miR-133 antagomir, MuRF-1 and atrogin1 exhibited a significant decrease (P<0.01) (Fig. 8m, n). Upon administration of miR-1+133 antagomir, there was a notable increase in Pax3, myf5 and MyoD proteins. It is suggested that injection of miR-1-3p antagonists primarily enhance muscle proliferation and differentiation, while injection of miR-133a-3p antagonists mainly promote differentiation and inhibit atrophy. Combined injection has demonstrated significant efficacy in promoting proliferation and differentiation.

## Discussion

Studies have demonstrated that miRNAs can induce significant alterations in the ultrastructure of skeletal muscle [22]. Following acute eccentric exercise, progressive changes in muscle ultrastructure were observed, with the most pronounced structural disruption occurring three days post-exercise and nearly complete recovery evident by one week. However, administration of miR-1-3p or miR-133a-3p antagonists immediately after acute eccentric exercise significantly ameliorated these exercise-induced ultrastructural abnormalities. Notably, in antagonist-treated rats, muscle ultrastructure had largely normalized by day three post-exercise – comparable to the condition observed in non-injected controls at one week. These findings suggest that miR-1-3p or miR-133a-3p antagonists effectively accelerate the repair of skeletal muscle fiber damage resulting from acute eccentric exercise.

Skeletal muscle injury regeneration is a highly intricate regulatory process. Following injury, resting muscle satellite cells are synchronously activated by Pax7 and subsequently induce downstream myogenic regulatory factors to facilitate proliferation and differentiation, thereby promoting the regeneration of muscle cells to repair damaged muscle fibers [23]. Immunostaining of Pax7 with the extracellular matrix protein laminin has revealed that satellite cells are promptly activated in response to exercise-induced muscle injury [24]. Consequently, there is a significant decrease in the number of resting muscle satellite cells immediately after exercise, followed by a substantial increase in their self-renewal and proliferation 12 h post-exercise. However, there is no significant change in the number of muscle satellite cells during the subsequent 24–72 h and one week after exercise, indicating that the muscles have gradually transitioned into stages of differentiation, fusion, and regeneration.

MRTF-A protein expression increased significantly at 12 h and 72 h post-exercise, whereas Pax7 and SRF protein expression decreased at 72 h and 1 week after exercise. As an upstream regulator of SRF and Pax7, MRTF-A not only synergistically promotes SRF expression to influence muscle differentiation [25], but also stimulates Pax7 expression through the MRTF-A/Pax7 axis to regulate muscle proliferation [8]. We found that the protein expression of Pax7 and SRF did not align with their respective mRNA trends. Protein levels were abnormally reduced at E72 hours and one week post-exercise despite sustained high mRNA levels. This implies potential involvement of miRNAs in regulating Pax7 and SRF, leading to translation inhibition and subsequent abnormal protein expression [26]. One week after the injury, the expression of MRTF-A protein returned to baseline levels, indicating that the damage has been partially rectified.

We found a significant increase in the expressions of miR-1 and miR-133 at 72 h post-exercise injury, indicating this as a specific time point for injury repair. The expression of miR-1-3p/miR-133a-3p was compared with the mRNA and protein levels of MRTF-A, Pax7, and SRF. The results indicated that the high expression of miR-1-3p/miR-133a-3p 72 h after exercise did not have an impact on the mRNA levels of these three genes. However, they potentially inhibited the translation of Pax7 and SRF proteins, and slightly reduced MRTF-A protein expression, suggesting a role for miR-1-3p/miR-133a-3p in the translational inhibition of Pax7, SRF, and MRTF-A. Studies have demonstrated that the *in vivo* administration of miRNA antagomir can effectively suppress miRNA activity, thereby enhancing the expression of target genes [22]. In this study, we confirmed that miR-1-3p and miR-133a-3p antagonists exert significant inhibitory effects. The levels of miR-1-3p and miR-133a-3p in all three injection groups were reduced to one-third or even lower than those in the negative control group, with the combined injection demonstrating the most potent inhibitory effect on these microRNAs. It is evident that a single injection of either miR-1-3p or miR-133a-3p can co-inhibit two miRNAs, possibly due to their co-transcription from the same chromosomal site during tissue-specific development [13], thereby producing a synergistic inhibition effect when targeted by antagonists.

In this study, it was observed that inhibition of miR-1a-3p led to increase in the protein expressions of MRTF-A, Pax7 and SRF. It suggested that miR-1a-3p may collectively target the 3′UTR region of MRTF-A, Pax7 and SRF to directly regulate their protein expression. SRF can directly induce the transcription of miR-1 in skeletal muscle precursor cells to promote skeletal muscle differentiation and regulate muscle hypertrophy [27]. In this study, the upregulation of SRF protein expression was observed following miR-1-3p inhibition, indicating that miR-1-3p may target SRF protein expression in rats. Furthermore, effective suppression of miR-133a-3p expression significantly enhanced Pax7 protein expression, consistent with the increase in Pax7/Laminin co-labeled muscle satellite cells. This suggests that injection of miR-133 antagonists can selectively promote Pax7 protein upregulation and increase muscle satellite cell numbers after acute eccentric exercise, thereby initiating a repair response and accelerating skeletal muscle regeneration. Following the inhibition of miR-133a-3p, there was an observed increase in SRF protein expression, indicating targeted inhibition by miR-133a-3p on SRF protein, consistent with previous findings [13]. Subsequent to the inhibition of miR-133a-3p, MRTF-A protein expression significantly increased, suggesting that miR-133a-3p also inhibits MRTF-A protein expression. Furthermore, we found that combined injection of miR-1-3p and miR-133a-3p antagonists resulted in the most significant increases in MRTF-A, Pax7 and SRF protein expressions; specifically doubling the expressions of MRTF-A and Pax7 along with Pax7-labeled satellite cells count. This is consistent with previous reports [28,29].

Pax7 initiates the activation of satellite cells to engage in proliferation, differentiation, and fusion processes following skeletal muscle damage, while also regulating downstream myogenic regulatory factors such as Myf5, MyoD, MyoG, and MRF4 [30]. In this study, both Pax3 and Myf5 exhibited a decrease immediately post-injury, followed by an increase and subsequent decrease during the regeneration phase. Pax3 expression preceded that of Myf5, with peak Pax3 protein levels observed 24 h post-sports injury, while peak Myf5 expression occurred 48 h post-injury, returning to pre-injury levels within one week. These findings are consistent with previous research [30,31]. A significant increase in MyoD protein expression was observed immediately post-injury, maintaining elevated levels until returning to baseline one week later. Furthermore, MyoG protein expression also showed a significant increase immediately after the injury, followed by a proliferative stage at 12–48 h post-injury. Subsequently, there was a notable decrease in MyoG protein expression which gradually increased again until 72 h and one week post-injury, consistent with previous research findings by Dimauro *et al*. [6]. The expression of MyoD preceded that of MyoG, consistent with the phasic changes in the proliferation and differentiation process. MyoD primarily plays a pivotal role in the late proliferative and early differentiation stages, while MyoG peaks during the differentiation stage. This indicates that skeletal muscle cells are in the early differentiation phase of repair and regeneration 72 h post-sports injury. After satellite cells are injured, MASTR and MRTF-A are upregulated, MASTR, as the main stimulator of skeletal muscle regeneration, activate the MyoD enhancer through its interaction with MEF2 and MRTF-A [8]. A trend of consistent protein expression between MRTF-A and MyoD was observed at 12–72 h and one week post-injury. This suggests that skeletal muscle cells are in the early differentiation stage of repair and regeneration 72 h after sports injury.

Previous studies have demonstrated that transfecting C2C12 myoblasts with miR-1 inhibitors can significantly enhance myoblast proliferation [13]. In this study, the miR-1-3p antagonists were also found to play a role in promoting proliferation, potentially mediated through the modulation of Pax3 and Myf5 protein expressions. It has been established that miR-133 can impede myocyte proliferation by activating cell cycle arrest genes [8], without impacting the secretion of proliferative factors Pax3 and Myf5. Consequently, the administration of miR-133a-3p antagonists did not have a significant impact on the protein expression of Pax3 and Myf5. The inhibition of the MyoD protein was only observed with miR-1-3p antagonists because miR-1-3p not only directly targets Pax7 to promote satellite cell proliferation, but also targets MRTF-A, leading to an increase in Pax7 expression through the MRTF-A/Pax7 axis and ultimately promoting the upregulation of Myf5. Moreover, miR-1-3p targets MRTF-A, thereby enhancing Pax7 expression through the MRTF-A/Pax7 axis and subsequently elevating Myf5. This collectively results in the inhibition of MyoD protein expression during early muscle differentiation [8], which aligns with the findings of Nakasa *et al*. in C2C12 myoblasts [32]. Notably, miR-133a can suppress both SRF and MRTF-A/SRF axis, whereas MRTF-A/SRF axis stimulates muscle differentiation by upregulating MyoD expression [8]. Therefore, the administration of a miR-133a-3p antagonist alone or in combination with a miR-1-3p antagonist did not significantly affect MyoD expression. The expression of MyoG protein was significantly upregulated 3 days post-exercise, and there was no significant alteration in MyoG protein levels following the injection of miR-1-3p and miR-133a-3p antagonists. However, the expression of MyoG protein was markedly increased after combined injection, indicating that the combined injection of miRNA antagonists had the most potent effect on inducing muscle differentiation. This finding is consistent with previous reports [33]. The E3 ubiquitin ligases muscle atrophy f-box (MAFbx, also known as atrogin-1) and muscle-specific ring finger protein 1 (MuRF-1), also known as TRIM63, are crucial regulators of ubiquitin-mediated protein degradation in skeletal muscle [34]. The expression of atrogin-1 and MuRF-1 is linked to the process of skeletal muscle injury [34]. We observed a notable rise in atrogin-1 protein levels immediately after the injury, which was then succeeded by a significant decline at 48 h and one week post-injury. These findings align with the results documented by Jensen *et al*. [36]. The expression of MuRF-1 protein was significantly elevated at 72 h and one week post-injury, displaying an opposite trend to Atrogin-1. It was reported that abundant MuRF-1 expression is associated with rapid activation of myogenesis after muscle injury, while relatively low MuRF-1 expression in dysproteinopathy may be attributed to impaired muscle regeneration [37]. In this study, the increased expression of MuRF-1 protein 72 h after injury indicates activation of postexercise myogenesis, which may promote the muscle regeneration process to some extent. Atrogin-1 protein expression remained largely unchanged following single or combined injections. However, MuRF-1 protein expression was notably suppressed by a single injection of miR-133a-3p antagonist. The expression of MuRF-1 protein was significantly upregulated at 3 days post-exercise, but there was no significant alteration following the injection of miR-1-3p antagonist and combined injection. It suggests that both interventions could also partially inhibit the expression of MuRF-1 protein, which could be beneficial for the muscle regeneration process following injury.

The study also presented certain limitations. Due to the skeletal muscle sports injury model, it was difficult to validate the function of miRNAs through the replication of a similar environment *in vitro* cell culture. Thus, we chose *in vivo* injection for validation. This study was restricted by only selecting miRNA antagonists for injection, without involving any agonists. Our study indicates that the optimal duration for a single injection of miRNA antagonists to repair exercise-induced skeletal muscle injury is three days. Further research is necessary to determine whether multiple injections are more effective before conducting clinical trials.

## Conclusions

The ultrastructure of the gastrocnemius in rats was significantly damaged after a single acute eccentric exercise, with the most severe injury occurring 72 h after the exercise. However, the damage was largely repaired within a week. The injection of miR-1-3p and miR-133a-3p antagonists immediately after eccentric exercise can significantly promote muscle proliferation and differentiation by targeting the MRTF-A/Pax7 axis and MRTF-A/SRF axis, inhibit muscle atrophy, and shorten the time required for muscle injury repair to three days. Compared with a single antagonist injection, the combined injection of the two is more effective.

## Figures and Tables

**Table 1:** Antibody information.

Antibody	Dilution	Brand	Cat. No.	Remarks
*Pax7 antibody*	1:50	STANTA CRUZ	sc-81648	IF
*Anti-Laminin*	1:50	Sigma	L9393	IF
*Anti-mouse IgG (H+L)Alexa Fluor®555 Conjugate*	1:1000	Cell Signaling	4409S	IF
*Anti-rabbit IgG (H+L)Alexa Fluor®488 Conjugate*	1:1000	Cell Signaling	4412S	IF
*Pax7 antibody*	1:1000	STANTA CRUZ	sc-81648	WB
*SRF (G93) polyclonal antibody*	1:1000	Bioworld Technology Inc	BS1332	WB
*MKL1 Rabbit pAb*	1:1000	ABclonal	A12598	WB
*Pax3 Polyclonal antibody*	1:1000	Proteintech	21386-1-AP	WB
*Myf5 monoclonal antibody*	1:2000	Bioworld Technology Inc	MB64096	WB
*MyoD1/Myf3 Polyclonal Antibody*	1:1000	Bioworld Technology Inc	BS65602	WB
*MyoG polyclonal antibody*	1:1000	Bioworld Technology Inc	BS8337	WB
*Fbx32 monoclonal antibody*	1:1000	Bioworld Technology Inc	BS65520	WB
*TRIM63 Rabbit pAb*	1:1000	ABclonal	A3101	WB
*GAPDH polyclonal antibody*	1:5000	Bioworld Technology Inc	AP0063	WB
*Tubulin α (G436) polyclonal antibody*	1:5000	Bioworld Technology Inc	BS1699	WB
*Goat anti-Rabbit IgG (H+L)-HRP*	1:10000	Bioworld Technology Inc	BS13278	WB
*Goat anti-Mouse IgG (H+L)-HRP*	1:10000	Bioworld Technology Inc	BS12478	WB

**Table 2:** Primers in the study.

Primer	Forward sequence (5′ to 3′)	Reverse sequence (5′ to 3′)
*Pax7*	GCGAGAAAGGCAACCG	TGCTCAGCCGTGAATGTG
*SRF*	AGAGGAAGACGGGCATCA	GCGGGTGGCAAAGGTAT
*MRTF-A*	CACCCACTCAGGTTCTTTCT	TTCGGTTGGCTTTGCTT
*β-actin*	GGGAAATCGTGCGTGAC	ACCCAGGAAGGAAGGCT
*miR-1-3p*	TGGAATGTAAAGAAGTGTGTAT	^/^ 1
*miR-133a-3p*	TTTGGTCCCCTTCAACCAGCTG	^/^ 1
*U6*	CTCGCTTCGGCAGCACA	AACGCTTCACGAATTTGCGT

1 The primer sequence is supplied as part of the kit configuration.
